# Correlation Analysis of Twig and Leaf Characteristics and Leaf Thermal Dissipation of *Hippophae rhamnoides* in the Riparian Zone of the Taohe River in Gansu Province, China

**DOI:** 10.3390/plants14020282

**Published:** 2025-01-20

**Authors:** Qun Li, Min Ma, Yurui Tang, Tingting Zhao, Chengzhang Zhao, Bo Li

**Affiliations:** 1 College of Resources and Environment, Xichang University, Xichang 615013, China; 2 School of Life Sciences, Fudan University, Shanghai 200433, China; 3 College of Geography and Environmental Science, Northwest Normal University, Lanzhou 730070, China; mamin@gsau.edu.cn (M.M.); 18419060862@163.com (Y.T.); zhtt123_56@163.com (T.Z.); 4 College of Geography and Environmental Science, Northwest Normal University, Research Center of Wetland Resources Protection and Industrial Development Engineering of Gansu Province, Lanzhou 730070, China

**Keywords:** twig diameter, leafing intensity, thermal dissipation, electron transport rate, *Hippophae rhamnoides*, riparian zone

## Abstract

**Aims**: The functional traits of twigs and leaves are closely related to the ability of plants to cope with heterogeneous environments. The analysis of the characteristics of twigs and leaves and leaf thermal dissipation in riparian plants is of great significance for exploring the light energy allocation and ecological adaptation strategies of plant leaves in heterogeneous habitats. However, there are few studies on the correlation between the twig–leaf characteristics of riparian plants and their heat dissipation in light heterogeneous environments. **Methods**: In this study, the riparian plant *Hippophae rhamnoides* in Taohe National Wetland Park was the research object. According to the differences in the canopy light environment of the *H. rhamnoides* population, three habitat gradients were set: I, the full sight zone; II, the moderate shade zone; and III, the canopy cover zone. We studied the relationship between the twig–leaf characteristics of *H. rhamnoides* and leaf thermal dissipation in a heterogeneous light environment. **Important Findings**: The results are as follows: from the full sight zone to the canopy cover zone, the population characteristics and the twig, leaf, and photosynthetic fluorescence physiological characteristics of *H. rhamnoides* demonstrated significant changes (*p* < 0.05). In the full sight zone, *H. rhamnoides* tended to have thick leaves with a smaller SLA on short and thick twigs, and the light energy absorbed by the leaves accounted for a higher proportion of thermal dissipation. In the moderate shade zone, *H. rhamnoides* tended to grow many thin leaves with high SLA on long and thick twigs, and the proportion of light energy absorbed by the leaves for heat dissipation was lower than that in the full sight zone. In the canopy cover zone, *H. rhamnoides* tended to grow a few large and thick leaves with a low SLA on slender and long twigs, and the proportion of light energy absorbed by the leaves for heat dissipation was the lowest. There was a significant correlation between the twig–leaf and leaf heat dissipation of *H. rhamnoides* in the three habitats (*p* < 0.05). The co-variation of plant branches and leaves and the timely adjustment of thermal dissipation in photoheterogeneous habitats reflect the phenotypic plasticity mechanism and self-protection strategy of riparian plants in adapting to heterogeneous environments.

## 1. Introduction

Plant functional traits are the survival, growth, and reproduction strategies of plants adapted to the external environment, which determine how plants respond to environmental changes and optimize resource utilization and reflect the ability of plants to obtain, utilize, and preserve resources [1,2,3]. Plants cope with environmental stress through the coordination or trade-off of traits in different environments so that the harm of adverse environmental factors to individual plants is minimized [4]. As one of the structural units of plants (twig, hereafter), the annual twigs of trees and shrubs are the most active part of the plant branching system [5,6], which can directly reflect the life history countermeasures of plants adapting to the environment. The leaves on the twigs are the main components of plants and the main organs for carrying out physiological activities and exerting ecological functions. Their structural traits, to a certain extent, determine the photosynthetic activity of plants and the optimization of resource utilization in response to environmental changes [7]. These traits can reflect the utilization level of the light energy absorbed by the leaves, which are also an organ with strong plasticity and sensitivity to environmental changes [8]. The combination of branches and leaves can better reflect plant configuration shaping and space resource utilization ability, which is one of the main dimensions of plant ecological strategy [9]. Branch structure affects leaf size and photosynthetic efficiency, which determines the trade-off between leaf size and leaf number, affects the carbon acquisition of plants, and further affects the growth and survival of branches [10]. The characteristics of leaves and branches are highly coordinated in biomechanics and the morphological structure, which determines the utilization of light, water, and other resources and the adaptation strategies of plants [11]. Therefore, the relationship between twigs and leaves can effectively explain the adaptation strategies of plants to the environment [12].

Light is one of the most important environmental factors affecting plant growth and distribution [13,14]. Shading is a common phenomenon among naturally growing plants that can reduce intense solar radiation, promote the accumulation of soil organic matter, reduce the amplitude of ambient temperature, etc., and plays an important regulatory role in building the optimal physiological and functional state of plants [15,16]. Long-term exposure to heterogeneous light distribution inhibited the availability of resources among plant populations [14], thus affecting plant growth and development and dry matter accumulation efficiency [17]. To ensure sufficient light energy capture, maintain photosynthesis, and achieve the maximum investment and return of photosynthesis products [18], plants preferentially allocate biomass to the trait with the easiest access to light resources [19,20] by adjusting the number leaves on twigs, single leaf area, leaf thickness, and other functional traits [21], thus promoting plants to weigh resource input among various components [22]. The change in the characteristics of twigs and leaves and the balanced distribution of biomass ensures the coordination and unity of the effectiveness and safety of water transport in the process of leaf photosynthesis [23,24].

Plant photosynthesis is a complex metabolic pathway that can reflect the adaptive countermeasures formed by plants to adapt to environmental changes and reflect the law of interaction between plants and the environment [25,26]. Chlorophyll fluorescence is closely related to the characteristic process of photosynthesis [26,27]. Part of the light energy absorbed by plant leaves is used in photochemical processes to synthesize sugars for life activities; the other part is dissipated in the form of fluorescence or heat to protect itself [28]. Chlorophyll fluorescence parameters reflect the changes in the process of plant photosynthesis, so it becomes an indicator of photosynthetic activity and behavior [29]. Non-photochemical quenching (NPQ) is considered the fastest process by which plants relieve the excitation energy pressure in the photosynthetic membrane (the site of the photosystem II reaction), thus protecting the plant from stress damage [30]. Light is the driver of photosynthesis in plants, but it also impairs the photosynthetic process in many ways [31]. In strong light environments, the non-photochemical quenching (NPQ) mechanism maintains the normal photosynthesis of plant leaves by dissipating excess light energy absorbed by plant leaves in the form of heat, which is an important protection mechanism of plant leaves [32]. The environmental resources (such as light, water, nutrients, etc.) received by individual plants in different habitats can significantly change the structural morphology and photosynthetic fluorescence characteristics of plant twigs and leaves [33]. In the shaded environment, the far red light is enriched [34,35,36], the photochemical efficiency of PSII is higher, the biomass distribution of plant branches and stems is increased, and the light distribution between the photosystems is conducive to PSII, which can promote photosynthesis and plant growth [37]. However, less attention has been paid to the correlation and response mechanism of chlorophyll fluorescence characteristics in plants in natural shade environments. Therefore, studies on the correlation between the twig–leaf traits and chlorophyll fluorescence characteristics of plants in natural shade environments can reflect the ecological strategies adopted by plants to cope with heterogeneous light environments and have important significance for understanding the mechanisms of phenotypic plasticity and self-protection of plants.

The riparian zone is the riverbed between high and low water levels and the area where the influence of the river transitions from the high water level to the river [38]. It is also an important ecological transition zone for the material, energy, and information exchange between the river and the terrestrial ecosystem [39]. It has rich biodiversity and provides an extremely wide range of ecosystem services and functions [40,41]. The seasonal and interannual changes in river hydrological conditions lead to the alternating cycle of flood and drought in riparian areas [39] and the changes in topography and soil structure. The particularity, complexity, and spatial heterogeneity of their habitats affect the resource acquisition and spatial distribution of riparian plant communities, thus changing the light, soil water, and nutrient resources available to populations within the communities. To improve the fitness of the population, the individual plants in the population can adjust their morphology and physiology [42,43]. *Hippophae rhamnoides* is a deciduous plant belonging to the Elaeagnaceae family, Hippophae. It grows rapidly, has strong root spreader ability, and has a wide range of adaptability. It is an excellent tree species for windbreak and sand fixing, soil and water conservation, and ecological environment improvement [44]. The strong habitat adaptability and environmental modification ability of *H. rhamnoides* can alleviate the impact of floods on the ground, create favorable conditions for the restoration of riparian biodiversity, and play a very important role in the survival and development of shoreline forests. At present, studies on *H. rhamnoides* mainly focus on leaf photosynthetic characteristics [45,46], morphological and physiological responses to salt stress [47], root studies [48], and ecological stoichiometry studies [49]. However, the correlation between the twig–leaf traits and photosynthetic fluorescence physiological characteristics of *H. rhamnoides* in heterogeneous habitats remains unclear.

Given this, this paper took *H. rhamnoides*, a riparian plant in the Taohe National Wetland Park, Gansu Province, as the research object and proposed the following hypothesis: *H. rhamnoides* would demonstrate adaptive changes in twig–leaf traits and photosynthetic fluorescence physiological characteristics in photoheterogeneous habitats. We aim to answer the following questions: (1) Under photoheterogeneous conditions, what is the change rule of the twig–leaf traits of *H. rhamnoides*? (2) What is the distribution of light energy absorbed by *H. rhamnoides* leaves in terms of photosynthesis and heat dissipation? What is the relationship between the twig–leaf characteristics of *H. rhamnoides* and leaf heat dissipation? This study helps shed light on the photosynthetic and photosynthetic fluorescence physiological processes of riparian plants in photoheterogeneous habitats and provides a scientific theoretical basis for the stable maintenance of riparian ecosystems and the management of riparian plants.

## 2. Materials and Methods

### 2.1. Study Sites

**Figure 1:**
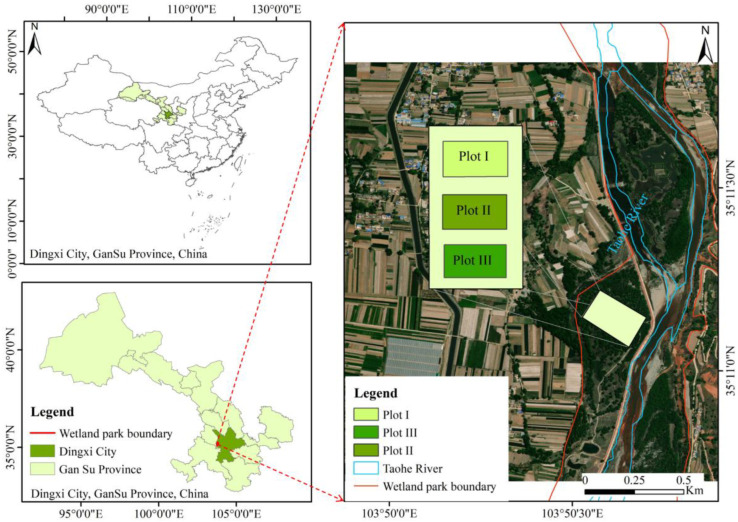
Study area and locations of the plots.

The study area is located in the south of Lintao County, Dingxi City, Gansu Province (103°45′43″–103°50′55″ E, 35°05′27″–35°15′58″ N; Figure 1), which is located in the intersection area of the Qinghai-Tibet Plateau and Loess Plateau. On the northwest margin of the Qinling fold system, there are undulating mountains in the east, west, and south and a deep gully with an altitude of 2200–2500 m. This region is a semi-arid and semi-humid climate zone in the middle and temperate zone. The average annual temperature is 7.2 °C, the average annual precipitation is 519.2 mm, and the annual solar radiation is 139.76 kCal/cm^2^. The Taohe River is a first-level tributary of the Yellow River, originating from the Henan Mongolian Autonomous County of Qinghai Province, flowing from the east through Luqu, Lintan, and Zhuoni County, south to Minxian County Chabu, and then turning to the northwest through Jiudian Gorge, Haidian Gorge, Lintao Basin, and Yongjing County, into the Liujiaxia Reservoir. The soil is mainly red soil, tidal soil, and sandy soil. The main plants are *H. rhamnoides*, *Periploca sepium*, *Populus simonii*, *Berberis thunbergii*, *Ligustrum lucidum*, and *Hedysarum multijugum* [50].

### 2.2. Experimental Methods and Design

Based on many field investigations and the accessibility of plant communities on both sides of the Taohe National Wetland Park, a 300 m × 200 m transect was set up along the river terrace in the middle part of the Taohe National Wetland Park from 20 to 31 August 2019, and a representative Qiaoguan community was selected from the transect. Due to the influence of canopy light resources, light acquisition by understory shrubs was limited, and the biological characteristics of *H. rhamnoides* populations were significantly different in various microhabitats. According to the canopy shelter and shrub distance of the *H. rhamnoides* population, the riparian tree–shrub community is divided into three light levels: plot I, the full sight zone, which is an open area more than 1 times the tree height from the forest margin; plot II, the moderate shade zone, is a forest clearance with a size of 1 times the height of the trees or a canopy gap with a diameter of not less than 5 m and direct light; plot III, the canopy cover zone, which is an environment in which the canopy is fully closed or intersecting with another canopy [44,51]. First, according to the habitat gradient of *H. rhamnoides*, six 5 m × 5 m quadrats were set for each light level, for a total of 18 quadrats. Then, plant community characteristics and the properties of the *H. rhamnoides* population (canopy density, plant height, crown width, and density) were recorded, and the altitude, longitude, and latitude of each quadrat were measured using a portable handheld GPS. Finally, six *H. rhamnoides* strains with good growth were selected as test plants in each quadrat, and the following indexes were measured.

#### 2.2.1. Measurement of the Leaf Photosynthetic Parameters of *H. rhamnoides*

From 09:00 to 12:00 on a sunny day, during the same period, from each *H. rhamnoides*, we selected four annual twigs from the middle and upper part of the canopy and marked them. The branch angle and leaf inclination angle of the twigs were measured with a protractor, and the average value was obtained. Two healthy, intact leaves were selected from the labeled twigs and wiped clean with dry gauze. The photosynthetic parameters were determined using a portable gas exchange system (GFS-3000 Heinz Walz GmbH, Effeltrich, Germany). During measurement, an artificial red–blue light source was used. The leaf portion inside the clamp-on leaf chamber was exposed to a photosynthetic photon flux density (PPFD) of 1200 µmol (photons) m^−2^ s^−1^. The experiments were performed outdoors with a controlled CO_2_ concentration (340 ± 10 μmol·mol^−1^), flow rate (750 ± 10 μmol·s^−1^), temperature (20 ± 5 °C), and humidity [relative humidity (RH) = 70 ± 5%]. The parameters of net photosynthetic rate *(P*_n,_) and transpiration rate (*T*_r_) were measured under an atmospheric CO_2_ concentration of 340 μmol·mol^−1,^ repeated three times for each marked *H. rhamnoides* plant. The water use efficiency is determined by calculating the *P*_n_/*T*_r_ ratio [52].

#### 2.2.2. Measurement of Photosynthetically Active Radiation (PAR) in the *H. rhamnoides* Population

During the same period of photosynthetic parameter determination, the photoquantum meter (3415F, Walz, Plainfield, IL, USA) was installed above the *H. rhamnoides* (15 cm from the canopy), in the middle (1/2 of the plant height), and on the surface (15 cm from the surface). The photosynthetically active radiation of *H. rhamnoides* was measured at 3 light levels [26].

#### 2.2.3. Measurement of Chlorophyll Fluorescence Parameters

Chlorophyll fluorescence parameters were measured using an IMAGING-PAM chlorophyll fluorescence system (Heinz Walz GmbH, Effeltrich, Germany) following a previously described method [53,54]. All chlorophyll fluorescence kinetics parameters were measured on the leaf after dark adaptation at ambient temperature for 30 min in each plot. The effective quantum yield of PSII photochemistry [Y(II)], the quantum yield of non-regulatory energy dissipation [Y(NO)], the quantum yield of regulatory energy dissipation [Y(NPQ)], photochemical quenching (QP), electron transport rate (ETR), and non-photochemical quenching (NPQ) were determined when steady-state chlorophyll fluorescence was attained after 5 min of actinic irradiance [26,52,55].

QP = (Fm′ − Fs)/Fv′ = 1 − (Fs − Fo′)/(Fm′ − Fo′)(1)

NPQ = (Fm − Fm′)/Fm′ = Fm/Fm′ − 1 (2)

Y(II) = ΦPSII = (Fm′ − Ft)/Fm′ (3)

Y(NO) = 1/(NPQ + 1 + qL (Fm/Fo − 1)) (4)

Y(NPQ) = 1 − Y(II) − Y(NO) (5)

ETR = 0.5 ×Y(II) × PAR × 0.84 (6)

Here, Fm′ is the maximum fluorescence under light, Fo′ is the minimum fluorescence under light, Fv′ is the fluorescence yield caused by saturated pulse light, Fs is steady-state fluorescence, Ft is real-time fluorescence, qL is the photochemical quenching coefficient based on the “lake model”, and PAR is photosynthetically active radiation [26].

#### 2.2.4. Measurement of Twig and Leaf (Twig Diameter, Twig Length, Number of Leaves, Fresh Leaf Weight, Leaf Area, Leaf Thickness, Leaf Dry Weight, Specific Leaf Area, and Chlorophyll Content) Traits of *H. rhamnoides*

The twigs with the epidermis of the mother branch after the measurement of leaf photosynthesis were cut and stored in a sealed plastic bag and brought back to the laboratory to complete the following measurements: First, the number of leaves on each twig was recorded, the leaves were removed, the fresh leaf weight was measured using an electronic balance (accuracy 0.0001 g) 3 times, and the average value was obtained. Then, a portable laser leaf area meter (CI-202, Walz, Camas, WA, USA) was used to determine the leaf area. The chlorophyll content was measured with a portable chlorophyll meter (SPAD-502, Minolta, Osaka, Japan) in different habitats [56]. The twig diameter and leaf thickness were measured using a vernier caliper with an accuracy of 0.02 mm. The leaf thickness was measured at different parts of the tested leaves, and the measurement was repeated 3 times to find the average value. The twig length was measured with steel tape (accuracy 0.1 mm). Finally, the leaves were put into an envelope, put into the oven at 105 °C for 30 min, and then dried at 85 °C until the constant weight was the dry weight of the leaves (accuracy 0.0001 g). The leaf water content was calculated using the following formula: (fresh leaf weight − dry leaf weight)/fresh leaf weight. SLA is expressed as the ratio of leaf area to leaf dry weight [26] (Li et al., 2022b). Leafing strength was calculated using the following formula: number of leaves/(length of twig × diameter of twig) [57].

#### 2.2.5. Determination of Soil Water Content, Soil Bulk Density, Soil Salinity, and the Contents of Sand, Silk, and Clay

First, a soil profile of 1 m × 1 m × 0.5 m was randomly excavated in the same square where the community investigation was conducted. Soil samples were taken with a ring knife (200 cm^3^) and divided into 5 layers 10 cm apart. The sample collection was repeated 3 times. Then, it was taken back to the laboratory to bake in the oven at 105 °C for 12 h. The mass was taken out and weighed, and the soil mass water content and soil bulk density of the 0–50 cm soil layer were calculated. The soil salt content was determined using the electrical conductivity method, and the electrical conductivity of the leaching solution was measured using a portable electrical conductivity meter (DDS-11C, Shanghai Lei Magnetic Instrument Factory, Shanghai, China). The average value was obtained after 3 repetitions. The mechanical composition of the soil was determined using the traditional screening method, and each soil sample was repeated three times. The weight of sand, silt, and clay were recorded separately (accuracy 0.0001 g). Finally, the proportion of sand, silt, and clay in each soil sample was calculated separately.

### 2.3. Statistical Analysis

All the original data of the experiment were sorted using Microsoft Excel 2021, and the water use efficiency, specific leaf area, and leafing intensity of *H. rhamnoides* were calculated. The biological traits and soil properties of the wetland communities in the three habitats were statistically analyzed. A one-way ANOVA was used to compare the twig–leaf characteristics, photosynthetic and fluorescent characteristics, and population traits of *H. rhamnoides* among the different habitats (α = 0.05). Then, R4.0.5 was used for a correlation analysis and mantel test. Other charts of the experiment were drawn using origin 2022.

## 3. Results

### 3.1. Physical and Chemical Properties of Soil with Different Habitats

**Figure 2:**
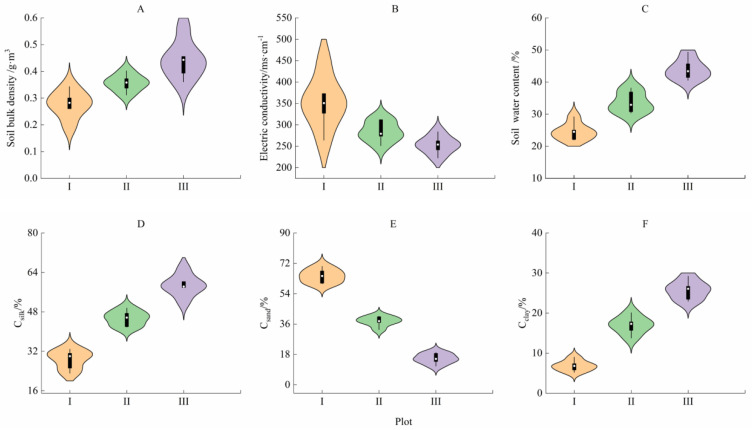
The physical and chemical properties of soil in each plot. I, Full sight zone; II, Moderate shade zone; III, Canopy cover zone. Different lowercase letters indicate significant differences among plots (*p* < 0.05). (**A**), the soil moisture content (SMC) in each plot; (**B**), soil electrical conductivity (EC) in each plot; (**C**), the soil bulk density (SBD) in each plot; (**D**), the content of silt (Csilt) in each plot; (**E**), the content of sand (Csand) in each plot; (**F**), the content of clay (Cclay) in each plot.

The physical and chemical properties of soil were significantly different in different habitats (Figure 2; Supplementary Table S1, *p* < 0.05). From the full sight zone (plot I) to the canopy cover zone (plot III), the soil bulk density, soil water content, soil silk content (Csilt), and soil clay content (Cclay) displayed increasing trends and increased by 79.09%, 59.89%, 1.04 times, and 2.77 times, respectively (A, B, D, F). The electric conductivity of the soil and the soil sand content (Csand) showed decreasing trends and decreased by 28.15% and 75.90%, respectively (B, E).

### 3.2. Biological Characteristics of Different Plant Community Habitats and Population Traits of H. rhamnoides

The photosynthetically active radiation (PAR) and crown density of the community were significantly different under different habitats (*p* < 0.05). From the full sight zone (plot I) to the canopy cover zone (plot III), the PAR of the community exhibited decreasing trends and decreased by 37.63%. The crown density displayed an increasing trend and increased by 1.42 times from the full sight zone to the canopy cover zone. Meanwhile, the population traits of *H. rhamnoides* were also significantly different from the full sight zone to the canopy cover zone. The crown width and average height and density of the *H. rhamnoides* population displayed increased trends, increasing by 1.67, 4.61, and 1.44 times, respectively (Table 1).

### 3.3. Leaf and Twig Traits of H. rhamnoides in Each Habitat

With the habitat change from the full sight zone to the canopy cover zone, the twig–leaf traits of *H. rhamnoides* were significantly different (*p* < 0.05; Table 2). The leaf water content and twig length displayed increasing trends and increased by 18.01% and 5.03%; meanwhile, the leaf area, leaf thickness, and chlorophyll content decreased first and then increased and ultimately increased by 1.83%, 6.78%, and 5.12%, respectively. Moreover, the specific leaf area showed the opposite trend and ultimately increased by 26.96%. The leaf inclination, branch angle, and leafing intensity displayed a decreasing trend and decreased by 62.17% and 17.12%, respectively; the twig diameter and the number of blades increased first and then decreased and ultimately decreased by 6.68% and 24.17%, respectively. The leaf fresh weight and leaf dry weight decreased first and then increased, and there was no significant change on the whole from the full sight zone to the canopy cover zone.

The plasticity index of the twig and leaf characteristics of *H. rhamnoides* was the leaf inclination, leaf dry weight, leaf fresh weight, specific leaf area, number of blades, twig diameter, leaf area, leaf intensity, branch angle, leaf thickness, leaf water content, chlorophyll content, and twig length in order from large to small.

### 3.4. Photosynthetic and Fluorescence Characteristics of H. rhamnoides in Each Habitat

The leaf fluorescence and photosynthetic characteristics of *H. rhamnoides* showed significant changes in different habitats (*p* < 0.05, Table 3). The actual photochemical efficiency of PSII (Y(II)) and photochemical quenching (QP) showed increasing trends from the full sight zone to the canopy cover zone and increased by 72.07% and 41.61%, respectively. The quantum yield of regulated energy dissipation (Y(NPQ)), the quantum yield of non-regulated energy dissipation (Y(NO)), and the net photosynthetic rate (*P*_n_) exhibited the largest values in the full sight zone and the lowest values in the canopy cover zone. From the full sight zone to the canopy cover zone, the Y(NPQ), Y(NO), and *P*_n_ decreased by 20.66%, 19.93%, and 25.30%, respectively. Meanwhile, the quantum yield of regulated energy dissipation (NPQ) and water use efficiency (WUE) increased first and then decreased and ultimately decreased by 5.28% and 22.70%, respectively. However, the electronic transport rate (ETR) presented the largest values in the moderate shade zone and the lowest values in the full sight zone and ultimately increased by 1.68 times. Moreover, the transpiration rate (*T*_r_) showed the lowest value in the moderate shade zone and the largest values in the canopy cover zone.

### 3.5. Conversion of Quantum Yields in PSII of H. rhamnoides Leaves in Each Habitat

**Figure 3:**
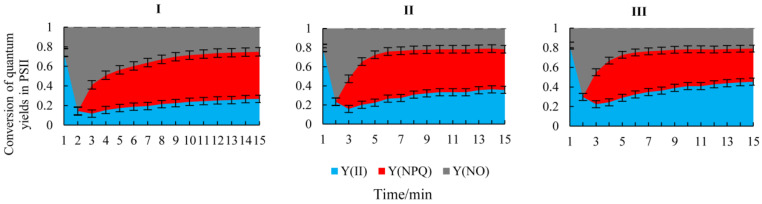
Conversion of quantum yields in photosystem II (PSII) in leaves of *H. rhamnoides* in each habitat (mean ± SD). Y(II), the photochemical quantum yield in PSII; Y(NO), the quantum yield of fluorescence and light-independent constitutive thermal dissipation; Y(NPQ), the quantum yield of thermal dissipation used in regulatory energy dissipation. Photosynthetically active radiation = 1200 μmol·m^−2^·s^−1^. (**I**), Full sight zone; (**II**), Moderate shade zone; (**III**), Canopy cover zone.

The proportion of actual photochemical efficiency of PSII (Y(II)) exhibited the largest values in the canopy cover zone and the lowest values in the full sight zone. The proportion of non-regulated energy dissipation (Y(NO)) presented the largest values in the full sight zone and the lowest values in the canopy cover zone. The quantum yield of regulated energy dissipation (Y(NPQ)) showed the largest values in the full sight zone and no significant changes from the moderate zone to the canopy cover zone. With the habitat change from the full sight zone to the canopy cover zone, the proportion of Y(NO) and Y(NPQ) decreased, whereas the proportion of Y(II) increased. The results indicated that the distribution of photosynthetically active radiation absorbed by *H. rhamnoides* leaves through photosynthesis, heat dissipation, and chlorophyll fluorescence changed significantly with the change in the light environment of the *H. rhamnoides* population (*p* < 0.05, Figure 3).

### 3.6. Changes in Electron Transfer Rate in H. rhamnoides Leaves in Each Habitat

**Figure 4:**
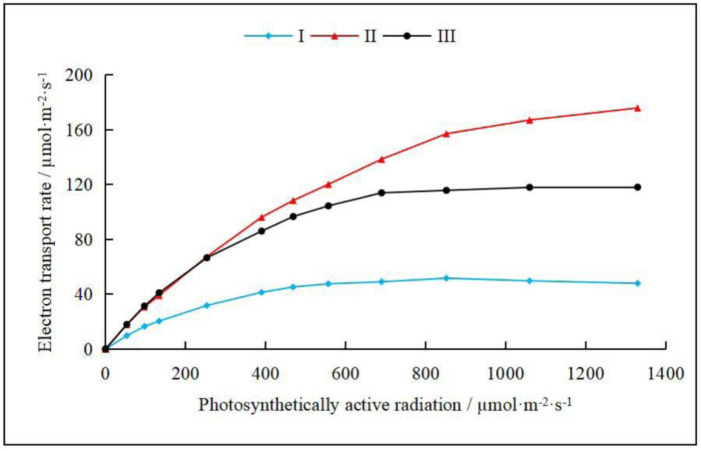
The electronic transport rate of *H. rhamnoides* leaves in each habitat (mean ± SD) I, I, Full sight zone; II, Moderate shade zone; III, Canopy cover zone.

There were significant differences in the fast optical response curves of the PSII electron transport rate in *H. rhamnoides* leaves in different light environments (*p* < 0.05, Figure 4). As the habitat changed from the full sight zone to the canopy cover zone, the electronic transport rate (ETR) of *H. rhamnoides* leaves showed the following trend: full sight zone (plot I) < canopy cover zone (plot III) < moderate shade zone (plot II). When the saturated pulse light intensity was higher than 1000 μmol·m^−2^·s^−1^, the ETR of *H. rhamnoides* leaves in Plot I and Plot II gradually changed from an increasing trend to a stable trend. However, when the saturated pulse light was stronger than 1200 μmol·m^−2^·s^−1^, the ETR of *H. rhamnoides* leaves showed a slow growth trend in the moderate shade zone. The results showed that the photosynthetic active radiation in the canopy of the plant community directly affected the ETR of *H. rhamnoides* leaves and then affected the photosynthesis of *H. rhamnoides* leaves. The ETR presented in plot I < plot III < plot II indicates that the moderate shade can promote electron transfer in riparian plant *H. rhamnoides* leaves to a certain extent.

### 3.7. Correlation Between the Twig–Leaf Characteristics and the Fluorescence Characteristics of H. rhamnoides

**Figure 5:**
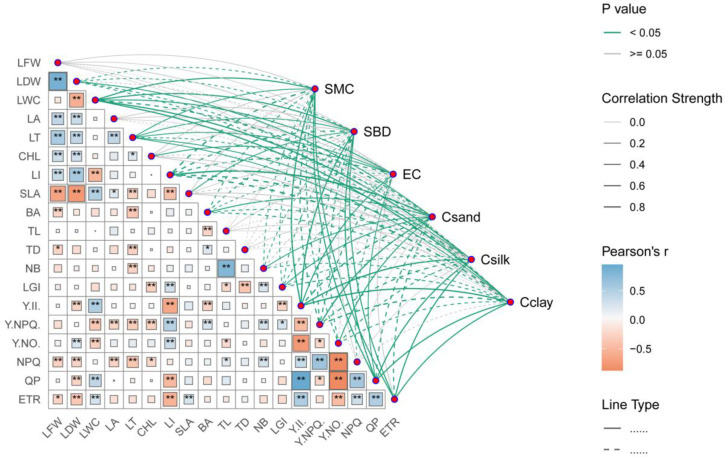
Correlation analysis between twig–leaf characteristics and fluorescence characteristics of *H. rhamnoides*. LFW, leaf fresh weight; LWC, leaf water content; CHL, chlorophyll content; LT, leaf thickness; LDW, leaf dry weight; LA, leaf area; LI, leaf inclination, SLA, specific leaf area; BA, branch angle; TL, twig length; TD, twig diameter; NB, the number of blade; Y(II), the actual photochemical efficiency of PSII; Y(NPQ), the quantum yield of regulated energy dissipation; Y(NO), the quantum yield of non-regulated energy dissipation; NPQ, the quantum yield of regulated energy dissipation; QP, photochemical quenching; T_r_, transpiration rate; *P_n_*, net photosynthetic rate; WUE, water use efficiency; ETR, electronic transport rate. * *p* < 0.05 (significant at the 0.05 level, bilateral; the null hypothesis is rejected at the 95% confidence level, and the sample shows a linear correlation); ** *p* < 0.01 (significant at the 0.01 level, bilateral; the null hypothesis is rejected at the 99% confidence level, and the sample shows a linear correlation). Blue represents a positive correlation between the twig–leaf characteristics and fluorescence characteristics, and tangerine represents a negative correlation between twig–leaf characteristics and fluorescence characteristics. The deeper the color, the more significant the correlation. The lighter the color, the weaker the correlation.

The Pearson correlation analysis results are shown in Figure 5 (Supplementary Table S2). There were highly significant negative correlations (*p* < 0.01, Figure 5, Supplementary Table S2) between the quantum yield of regulated energy dissipation (NPQ) and leaf fresh weight (LFW), leaf dry weight (LDW), leaf area (LA), and leaf thickness (LT). Meanwhile, the same correlation was observed between the proportion of the actual photochemical efficiency of PSII (Y(II)) and LDW, leaf inclination (LI), branch angle (BA), and leafing intensity (LGI), as well as between the quantum yield of regulated energy dissipation (Y(NPQ)) and leaf water content (LWC), LA, LT, and CHL, the proportion of non-regulated energy dissipation (Y(NO)) and LWC and LI, and the photochemical quenching (QP) and LDW and LI. In addition, there was a less significant negative correlation (*p* > 0.05, Figure 5, Supplementary Table S2) between NPQ and CHL and between Y(NO) and twig length (TL) in the three habitats. Moreover, there were highly significant positive correlations (*p* < 0.01, Figure 5, Supplementary Table S2) between Y(II) and LWC, as well as between Y(NO) and LDW. In addition, the same correlations were detected between Y(NPQ) and LI, BA, and the number of blades (NB); between NPQ and NB; and between QP and LWC. In addition, there was a less significant positive correlation (*p* > 0.05, Figure 5, Supplementary Table S2) between NPQ and twig length (TL) and LGI among the three habitats.

The mantel test showed that the soil moisture content (SMC), the content of silt (Csilt), the content of clay (Cclay), and the soil bulk density (SBD) were significantly correlated with Y(II), LWC, QP, ETR, LA, and leaf length, while the content of sand (Csand) was significantly correlated with Y(NPQ), WUE, and leaf inclination (LI). Moreover, soil electrical conductivity (EC) was significantly correlated with Y(II) and Tr. There is also a significant correlation between the fluorescence characteristics of *H.rhamnoides* and the six environmental factors in three density gradients (Figure 5; Supplementary Table S2).

## 4. Discussion

The functional traits of branches and leaves determine how plants respond to environmental changes and optimize resource utilization [7]. They are organs that are more sensitive to environmental changes and have strong plasticity [8], and they are also the main organs and energy converters for plants to obtain carbon through photosynthesis. In this study, it was found that the number of leaves, leaf inclination, branch angle, Y(NPQ), Y(NO), NPQ, Pn, and WUE of *H. rhamnoides* gradually decreased with the change in the light environment from the full sight zone (I) to the canopy cover zone (III). Leaf water content, leaf thickness, specific leaf area, chlorophyll content, Y(II), QP, Tr, and ETR were increased overall and tended to form thin and long twigs (Table 2 and Table 3). There was a significant correlation between the twig–leaf and leaf fluorescence parameters of *H. rhamnoides* (*p* < 0.05, Figure 5, Supplementary Table S2).

### 4.1. Correlation Between the Twig–Leaf Characteristics and the Fluorescence Characteristics of H. rhamnoides in the Full Sight Zone

The resource allocation and configuration characteristics of twigs are important topics in the study of plant life history strategies [58]. In the full sight zone, the photosynthetically active radiation was the highest, the canopy density of the plant community was the lowest, and the crown width, average height, and density of the *H. rhamnoides* population were the lowest (Table 1). The soil water content, bulk density, and proportion of silt and clay particles were the smallest, and the soil electrical conductivity and sand content were the highest (Figure 2; Supplementary Table S1). In this habitat, *H. rhamnoides* tended to be built on short and thick twigs, with thicker leaves and less SLA. The light energy absorbed by the leaves also accounted for a higher proportion of heat dissipation, and there was a significant correlation between the twig–leaf traits and chlorophyll fluorescence parameters (*p* < 0.05, Figure 5, Supplementary Table S2). The main reasons are as follows: (1) The habitat had abundant light resources, low soil bulk density and water content, high soil salinity and sand content, and poor soil water holding capacity. Compared with large leaves, small leaves with a low SLA have stronger thermal conductivity and smaller transpiration areas and can accommodate more chloroplasts, which is conducive to the adaptation of *H. rhamnoides* to a strong light environment [59,60]. While ensuring the effective capture of light energy, it can prevent water loss of leaves caused by overheating in strong light environments so that *H. rhamnoides* can maintain a high water use efficiency (Table 3) and avoid the phenomenon of holes and embolism caused by *H. rhamnoides* in strong light environments. Therefore, in this habitat, *H. rhamnoides* did not need to build a large photosynthetic area and formed a low-SLA strategy by reducing the leaf area to adapt to the strong light environment. (2) Under the combined stress of water and salt, the effective supply of water resources is insufficient, and the short and thick twigs shorten the effective transport distance of water from the root system to the leaf of *H. rhamnoides* [61], enhancing the water transport ability and maintaining the water transportation in *H. rhamnoides*. This is basically consistent with the research results of Wang et al. [62], which state that “water stress will significantly limit the longitudinal growth of plants while having little effect on radial growth, which is beneficial for increasing water utilization efficiency”. (3) In the full sight zone, *H. rhamnoides* had the lowest electron transfer rate (Figure 4). In order to prevent excessive light from damaging the photosynthetic structure of its leaves, *H. rhamnoides* used the acquired light energy for heat dissipation, and Y(NPQ) was the largest. Y(II) is the smallest (Table 3; Figure 3). This heat dissipation ability of leaves helps to prevent excessive water loss in plant leaves and improve plant adaptation to high-light habitats [59,60]. Therefore, the leaf heat dissipation of *H. rhamnoides* was significantly negatively correlated with leaf area and leaf thickness (*p* < 0.05, Figure 5, Supplementary Table S2).

### 4.2. Correlation Between the Twig–Leaf Characteristics and Fluorescence Characteristics of H. rhamnoides in the Moderate Shade Zone

Annual branches are the most dynamic part of the plant body, which is the support body of the leaves that determines the spatial distribution of the leaves. These branches allow the plant body to make full use of light energy for photosynthesis and reflect the response of the plant to the environment better than the whole plant can [63]. In the moderate shade zone, the SMC and SBD were higher than that in the full sight zone but lower than the canopy cover zone; the EC, Csilk, and Cclay were higher; and the Csand was lower than that in the full sight zone (Figure 2; Supplementary Table S1). The photosynthetic active radiation was lower than that in the full sight zone, the canopy density of the plant community was higher than that in the full sight zone, and the crown width, average height, and density of *H. rhamnoides* population were higher than that in the full sight zone (Table 1). In this habitat, *H. rhamnoides* tended to grow many thin leaves with high SLA on long and thick twigs, and the proportion of light energy absorbed by the leaves for heat dissipation was lower than that in the full sight zone. There was a significant correlation between the twig–leaf traits and chlorophyll fluorescence parameters (*p* < 0.05, Figure 5, Supplementary Table S2). The main reasons are as follows: (1) in the moderate shade zone, *H. rhamnoides* is in the excessive zone of the strong and weak light environments and has low survival pressure. To avoid the risk of water stress caused by excessive transpiration due to excessive leaf area, *H. rhamnoides* only needs to slowly increase the photosynthetic area to meet its growth demand. Therefore, it has a small Pn (Table 3). The investment of *H. rhamnoides* in leaf resources in this habitat was smaller than that in the strong light environment, resulting in thin leaves with high SLA, which helped to increase the effective utilization of interforest light resources by *H. rhamnoides* and maximize leaf WUE (Table 3). (2) In this habitat, the hydrothermal conditions were moderate, the soil water holding capacity was good, and there were no obvious resource constraints. *H. rhamnoides* was an associated species. To improve the interspecific competitiveness of *H. rhamnoides*, large and thin leaves were grown on long and thick twigs with the largest branch angle (Table 2). This could not only maintain a higher water supply capacity in the leaves but could also increase the vertical light resource competition ability of the *H. rhamnoides* population to maintain higher photosynthetic efficiency. (3) Chlorophyll fluorescence is closely related to the photosynthetic process of leaves [26,27,46]. In this habitat, the light intensity was moderate, and the proportion of light energy absorbed by *H. rhamnoides* leaves for heat dissipation decreased in the full sight zone. In addition, Y(NPQ) was lower, which increased the proportion of energy used for photosynthesis, and Y(II) was higher (Table 3; Figure 3). The decrease in light intensity was accompanied by a decrease in the light inhibition, heat dissipation, and transpiration water consumption of leaves, which resulted in an increase in the electron transfer rate of *H. rhamnoides* leaves (Table 3; Figure 4). *H. rhamnoides* tablets maintained a high water use efficiency (Table 3), thereby ensuring the smooth reproduction and renewal of the *H. rhamnoides* population.

### 4.3. Correlation Between the Twig–Leaf Characteristics and Fluorescence Characteristics of H. rhamnoides in the Canopy Cover Zone

The degree of crowding and resource competition within the population was high, and the available space occupied by leaves was relatively narrow [64]. In the canopy cover zone, the SMC, SBD, Csilk, and Cclay were highest, and the Csand and EC were lowest (Figure 2; Supplementary Table S1). The photosynthetic active radiation was the lowest, the vegetation community was the highest, and the crown width, average height, and density of the *H. rhamnoides* population were the highest (Table 1). In this habitat, *H. rhamnoides* tended to grow large and thick leaves with a small amount of SLA on long and thin twigs, and the proportion of light energy absorbed by leaves for heat dissipation was the lowest. There was also a significant correlation between the twig–leaf and chlorophyll fluorescence parameters (*p* < 0.05, Figure 5, Supplementary Table S2). The main reasons are as follows: (1) In the canopy cover zone, the soil water content was sufficient. The primary problem *H. rhamnoides* faced was the limitation of light resources caused by the shading of poplar and other trees in the canopy layer. To improve the competitiveness of light resources, *H. rhamnoides* formed thick leaves with a high SLA. The water content and chlorophyll content of leaves were increased (Table 2), and the normal photosynthetic physiological process of *H. rhamnoides* was maintained. This is different from the large and thin leaves formed by reeds under low light conditions reported by Li et al. [26], mainly because the leaf type of *H. rhamnoides* is different from that of reed leaves. (2) This habitat had the largest canopy density, and the construction of thin and long twigs was conducive to improving the interspecific competitiveness of *H. rhamnoides.* Placing the leaves on the twig at a higher vertical position was conducive to the acquisition and utilization of limited light resources by *H. rhamnoides.* The higher chlorophyll content in thick leaves maintained a higher electron transfer rate in *H. rhamnoides* leaves. However, compared with the moderate shade zone and full sight zone, a higher electron transfer rate in *H. rhamnoides* leaves was observed. The transpiration rate, net photosynthetic rate, and water use efficiency of *H. rhamnoides* leaves were significantly decreased (Table 3). (3) Chlorophyll fluorescence is the fastest and most efficient way to perceive external environmental stress [26]. In the canopy cover zone, light intensity decreased significantly, and *H. rhamnoides* leaves were exposed to weak light stress. Therefore, there was no need to use too much absorbed light energy for leaf heat dissipation, and Y(NPQ) and NPQ were the lowest, but the photosynthetic energy proportion was increased. Qp and Y(II) were the highest (Table 3; Figure 3). The results showed that *H. rhamnoides* PSII had higher photochemical efficiency in shaded environments [35,36], reflecting the phenotypic plasticity mechanism of *H. rhamnoides,* which adjusts the morphology structure and physiological characteristics of the twigs–leaves in time with the change in habitat conditions.

## 5. Conclusions

This study found that in the full sight zone, *H. rhamnoides* built thick leaves with a smaller SLA on short and thick twigs. A relatively high proportion of the light energy absorbed by the leaves was used for heat dissipation, which was an effective way for *H. rhamnoides* to adapt to the strong light environment. In the moderate shade zone, *H. rhamnoides* tended to grow many thin leaves with high SLA on long and thick branchlets, and the proportion of light energy absorbed by the leaves for heat dissipation was low. In the canopy cover zone, *H. rhamnoides* tended to grow a few large and thick leaves with a low SLA on slender and long twigs. The proportion of the light energy absorbed by the leaves used for heat dissipation was the lowest. This is a survival strategy of *H. rhamnoides* that enhances interspecific competition, alleviates intraspecific resource competition, and allows the plant to adapt to the weak light environment. In heterogeneous light habitats, the coordinated variation of the plant’s twig–leaf traits and the timely adjustment of the leaf photosynthetic fluorescence enhance the fitness of riparian plants, reflecting the ecological adaptation strategies of plants to improve interspecific competition and maintain normal photosynthetic physiological processes within the population. This study only analyzed the response patterns of twig–leaf traits and photosynthetic fluorescence physiological characteristics to the light environment from the perspective of light resources. The functional traits of other plant components in the riparian zone also play a crucial role in responding to heterogeneous environments, and we will conduct further exploration and research on this topic.

## Figures and Tables

**Table 1:** Community and population traits of *H. rhamnoides* in different plots (mean ± SE).

Plot	Community Traits		Population Traits		
	PAR/μmol·m^−2^·s^−1^	Crown Density/%	Crown Wide/cm	Average Height/cm	Density/Strain·m^2^
I	1457.33 ± 45.41 a	58.33 ± 4.65 c	55.50 ± 9.86 c	40.00 ± 5.99 c	20 ± 0.92 c
II	1167.33 ± 38.32 b	90.88 ± 6.84 b	99.80 ± 9.31 b	103.75 ± 4.62 b	29 ± 1.25 b
III	909.00 ± 22.35 c	141.02 ± 15.00 a	148.10 ± 10.12 a	216.58 ± 14.74 a	48 ± 2.42 a

Different lowercase letters in the same column indicate significant differences among plots (*p* < 0.05, n = 36). I, Full sight zone; II, Moderate shade zone; III, Canopy cover zone.

**Table 2:** Main leaf and twig characteristics of *H. rhamnoides* in each habitat (mean ± SE).

Plot	I	II	III	Plasticity Index
Leaf fresh weight/g	0.05 ± 0.002 a	0.03 ± 0.001 b	0.05 ± 0.001 a	0.52
leaf dry weight/g	0.02 ± 0.001 a	0.01 ± 0.001 b	0.02 ± 0.001 a	0.58
leaf water content/%	57.15 ± 1.13 c	63.86 ±1.01 b	67.45 ± 0.73 a	0.15
leaf area/cm^2^	1.98 ± 0.08 a	1.49 ± 0.07 b	2.02 ± 0.07 a	0.26
leaf thickness/mm	0.16 ±0.002 b	0.14 ± 0.002 c	0.17 ± 0.003 a	0.18
Chlorophyll content/SPAD	63.16 ± 1.87 b	58.59 ± 0.99 c	66.39 ± 1.10 a	0.12
leaf inclination/°	82.78 ±1.79 a	32.50 ± 0.68 b	31.31 ± 0.65 c	0.62
specific leaf area/cm^2^·g^−1^	98.74 ± 5.64 c	177.11 ± 11.17 a	125.36 ± 6.62 b	0.44
Branch Angle/°	74.02 ± 2.25 a	76.82 ± 1.66 a	61.35 ± 2.44 b	0.20
Twig length/cm	9.26 ± 0.58 a	9.50 ± 0.57 a	9.74 ± 0.70 a	0.05
Twig diameter/cm	1.66 ± 0.04 b	2.12 ± 0.20 a	1.55 ± 0.05 c	0.27
Number of blades/NO.	26 ± 2 a	28 ± 2 a	20 ± 2 b	0.30
Leafing intensity	1.69 ± 0.08 a	1.56 ± 0.08 a	1.32 ± 0.06 b	0.22

Different lowercase letters in the same row indicate significant differences among plots (*p* < 0.05, n = 36). I, Full sight zone; II, Moderate shade zone; III, Canopy cover zone.

**Table 3:** Photosynthetic and fluorescence characteristics of *H. rhamnoides leaves* in each habitat (mean ± SE).

Plot	I	II	III
Y(II)	0.22 ± 0.01 c	0.31 ± 0.01 b	0.40 ± 0.01 a
Y(NPQ)	0.46 ± 0.01 a	0.44 ± 0.01 a	0.37 ± 0.01 b
Y(NO)	0.32 ± 0.02 a	0.25 ± 0.01 b	0.25 ± 0.01 b
NPQ	0.40 ± 0.02 b	0.47 ± 0.02 a	0.38 ± 0.01 c
Photochemical quenching	0.41± 0.02 c	0.53 ± 0.01 b	0.59 ± 0.02 a
Electronic transport rate/µmol·m^−2^·s^−1^	37.08 ± 2.50 c	105.75 ± 10.71 a	99.3 ± 8.51 b
Transpiration rate/mmol H_2_O·m^−2^·s^−1^	1.18 ± 0.01 b	1.04 ± 0.04 c	1.21 ± 0.10 a
Net photosynthetic rate/μmol CO_2_·m^−2^·s^−1^	7.53 ± 0.03 a	7.16 ± 0.24 a	5.63 ± 0.48 b
Water use efficiency/μmol CO_2_·mmol H_2_O^−1^	6.41 ± 0.09 b	6.90 ± 0.11 a	4.95 ± 0.20 c

Different lowercase letters in the same row indicate a significant difference among plots (*p* < 0.05. n = 36). I, Full sight zone; II, Moderate shade zone; III, Canopy cover zone. Y(II), the actual photochemical efficiency of PSII; Y(NPQ), the quantum yield of regulated energy dissipation; Y(NO), the quantum yield of non-regulated energy dissipation; NPQ, the quantum yield of regulated energy dissipation.

## Data Availability

The data used in the present work have been listed in the Supplementary Materials.

## Supplementary Materials

The following supporting information can be downloaded at: https://www.mdpi.com/article/10.3390/plants14020282/s1, Table S1: The physical and chemical properties of soil in different plot (mean ± SE); Table S2: Correlation analysis between twig-leaf traits and fluorescence characteristics of *H. rhamnoides*.
