# Supplementary material for: Correlation Analysis of Twig and Leaf Characteristics and Leaf Thermal Dissipation of Hippophae rhamnoides in the Riparian Zone of the Taohe River in Gansu Province, China
Source: Plants (Basel). 2025 Jan 20;14(2):282. doi: 10.3390/plants14020282 (PMC11769175; doi:10.3390/plants14020282)
Supplement: Supplementary file 1 [file plants-14-00282-s001.zip › plants-3393648-supplementary.pdf]

Supporting information

Table S1. The physical and chemical properties of soil in different plot (mean ± *SE*)

Plot	SMC/%	SBD/g ·cm^{-3}	EC/μs·cm	Csand/%	Csilk/%	Cclay/%
I	24.54 ± 1.07c	0.28± 0.02c	352.17±24.41a	64.45± 1.65a	28.72±1.53c	6.83± 0.53c
II	33.67± 1.31b	0.36± 0.01b	284.51± 9.67b	37.65± 1.17b	45.36± 1.22b	17.00± 0.87b
III	43.96± 1.30a	0.44± 0.03a	253.04±8.35 c	15.53±1.22 c	58.73± 1.74a	25.74± 0.93a

Different lowercase letters in the same column indicate significant differences among plots ($p < 0.05$). I,Full sight zone; II, Moderate shade zone; III, Canopy cover zone. Csand, the content of sand; Csilk, the content of silk; Cclay, the content of clay; SMC, soil moisture content; EC, Soil electrical conductivity; SBD, Soil bulk density.

Table S2. Correlation analysis between twig-leaf traits and fluorescence characteristics of *H.rhamnoides*

	LFW	LDW	LWC	LA	LT	CHL	LI	SLA	BA	TL	TD	NB	LGI	Y(II)	Y(NPQ)	Y(NO)	NPQ	QP	ETR
LFW	1																		
LDW	.865**	1																	
LWC	-.098	-.565**	1																
LA	.403**	.344**	-.029	1															
LT	.549**	.403**	.044	.400**	1														
CHL	.327**	.299**	-.066	.176	.205*	1													
LI	.357**	.562**	-.501**	.164	.082	.002	1												
SLA	-.651**	-.750**	.490**	.205*	-.289**	-.181	-.416**	1											
BA	-.248**	-.101	-.169	-.119	-.363**	-.010	.153	.094	1										
TL	.029	.021	.002	.179	-.034	.112	-.045	.067	-.252**	1									
TD	-.243*	-.182	-.039	-.181	-.270**	-.012	-.096	.048	.222*	.072	1								
NB	-.160	-.071	-.098	.036	-.268**	-.092	.111	.142	-.049	.787**	.138	1							
LGI	-.076	.041	-.180	-.103	-.103	-.281**	.293**	-.025	.080	-.214*	-.375**	.251**	1						
Y(II)	.031	-.257**	.510**	.054	.067	.065	-.628**	.187	-.266**	.150	-.069	-.064	-.259**	1					
Y(NPQ)	-.170	.048	-.357**	-.254**	-.317**	-.288**	.473**	-.129	.259**	.049	.154	.262**	.246*	-.469**	1				
Y(NO)	.094	.248**	-.294**	.130	.164	.144	.337**	-.110	.098	-.201*	-.039	-.126	.100	-.750**	-.233*	1			
NPQ	-.278**	-.263**	.052	-.262**	-.365**	-.240*	-.090	.117	.111	.194*	.155	.269**	.044	.356**	.622**	-.858**	1		
QP	-.033	-.255**	.411**	-.005	-.053	-.012	-.484**	.171	-.174	.186	-.038	.045	-.175	.951**	-.229*	-.875**	.582**	1	
ETR	-.243*	-.359**	.281**	-.166	-.152	-.132	-.538**	.259**	-.091	-.088	.097	-.157	-.187	.520**	-.142	-.465**	.321**	.508**	1

LFW, leaf fresh weight; LWC, leaf water content; CHL, Chlorophyll content; LT, leaf thickness; LDW, Leaf dry weight; LA, leaf area; LI, leaf inclination, SLA, specific leaf area; BA, branch angle; TL, Twig length; TD, Twig diameter; NB, the number of blade; Y(II), the actual photochemical efficiency of PSII; Y(NPQ), the quantum yield of regulated energy dissipation; Y(NO), the quantum yield of non-regulated energy dissipation; NPQ, the quantum yield of regulated energy dissipation; QP, Photochemical quenching; Tr, Transpiration rate; Pn, Net photosynthetic rate; WUE, Water use efficiency; ETR, Electronic transport rate.* $P < 0.05$ (significant at the 0.05 level, bilateral; the null hypothesis is rejected at the 95% confidence level, and the sample shows a linear correlation); ** $P < 0.01$ (significant at the 0.01 level, bilateral; the null hypothesis is rejected at the 99% confidence level, and the sample shows a linear correlation)
